# The Effects of Physical Activity on Positive Emotions in Children and Adolescents: A Systematic Review and Meta-Analysis

**DOI:** 10.3390/ijerph192114185

**Published:** 2022-10-30

**Authors:** Jiayu Li, Zan Huang, Wenna Si, Tianyi Shao

**Affiliations:** 1 College of Physical Education and Health Sciences, Zhejiang Normal University, Jinhua 321004, China; 2 College of Teacher Education, Zhejiang Normal University, Jinhua 321004, China

**Keywords:** physical activity, children and adolescents, positive emotions, intervention studies, meta-analysis

## Abstract

Background: Positive emotions help children and adolescents develop good personalities and interpersonal relationships. Evidence shows that participation in physical activity is associated with positive emotions in young people. However, there is still a lack of studies on the effects of physical activity on positive emotions in children and adolescents. Objective: The purpose of this systematic review and meta-analysis was to examine the effects of physical activity on positive emotions in children and adolescents. Methods: Online databases (Scopus, PubMed, Web of Science, EBSCOhost, and APA PsycInfo) were searched from inception to August 2022. Result: A total of 24 articles were eventually included representing 3907 participants from 14 different countries. Overall, the effect of physical activity interventions on positive emotions was significant. The studies revealed that positive emotions were statistically better in the physical activity participation group than in the control group without physical activity (SMD = 0.62, 95% CI: (0.24, 1.01), *(p* < 0.01). Based on subgroup analyses, we found that participation in aerobic exercise for 30–60 min in adolescents aged ≥12 years had a more significant intervention effect on positive emotions. Conclusion: These findings indicate that the moods of children and adolescents who participate in physical activity significantly improved compared with the moods of those who do not participate in physical activity. The age, exercise type, and exercise duration of adolescents are important factors influencing the positive emotions resulting from physical activity interventions.

## 1. Introduction

Emotions are subjective internal human experiences expressed in facial expressions and physical movements, and they are classified under the umbrella of mental health [1]. Positive emotions, as positive components of mental well-being, are generally characterized by joy, enjoyment, and contentment, reflecting an individual’s overall subjective well-being [2,3]. Childhood and adolescence are considered the ideal time in life to encourage the development of positive emotions. Positive emotions help children and adolescents develop favorable personalities [4], organize interpersonal relationships [5], and solve group-related problems. This helps them to maintain vitality for a greater sense of well-being [6]. However, the emotions of children and adolescents are not stable or constant during their development. A study showed that emotions show dynamic and systematic changes throughout childhood and adolescence. In particular, in late adolescence, the stability of positive emotions is reduced [7].

Evidence has been presented that the emotions of children and adolescents are influenced by genetic, physiological, environmental, and life factors [8,9,10]. Although humans can adjust their emotions, external interventions are necessary. As children and adolescents are in a particular period of growth and development, the function of their brain organs is not yet complete [11]. Educators and psychologists have attempted to enhance positive emotions in children and adolescents in various ways, such as via music, painting, and literature [12,13]. However, with the prevalence of electronic devices and sedentary behavior, the above interventions are of limited effectiveness. A large number of children and adolescents have changed their lifestyles, and consequently, the emotional problems of children and adolescents have become increasingly serious [14]. Not only do negative emotions in children and adolescents disrupt social relationships, but they may also lead to lower academic achievement, dealing a double blow to well-being [15]. Therefore, in order to improve positive emotions in young people, scholars are focusing on sports as a special tool.

In recent years, articles on physical activity interventions and positive emotions have shown that the duration of sports intervention may have different effects on emotions. Sustained and appropriate physical activity may alter the structure and function of the brain. It has been found that physical activity improves emotions by increasing brain concentrations of dopamine, serotonin, and norepinephrine [16]. Excessive physical activity may trigger the production of androgenic anabolic steroids, leading to a significant increase in irritability and aggression, potentially triggering negative emotions [17]. These are related to neurobiological mechanisms in humans. The type of exercise also possibly influences emotion. Balchin’s study showed that high-intensity aerobic exercise was positively associated with a positive mood [18]. Cassilhas’s study showed that moderate-intensity anaerobic exercise leads to a greater improvement in the mood [19]. Some studies have also shown no clear relationship between exercise type and changes in the mood [20,21]. These different findings have stimulated our research interest. Three articles were retrieved on the association of physical activity participation with positive emotions. Zhang’s review (2019) found that aerobic and stretching/balancing exercises effectively improved well-being. However, the study looked at “older adults and cancer survivors” [22]. Rodriguez-Ayllon’s review (2019) examined the effects of physical activity and sedentary behavior on the mental health of children and adolescents. The review showed that physical activity is correlated with mental illness (i.e., negative effects, such as depression, anxiety, and stress) and mental health (i.e., self-esteem, self-concept, self-efficacy, optimism, and happiness), but it did not focus on “positive emotions” [23]. A review by Cho (2020) showed that school physical education program interventions improved mental health in adolescents [24]. The review studied adolescents involved in classroom physical activities. It mentioned mental health but did not focus on the theme of “positive emotions”. Considering these characteristics, this study focused on the keyword "positive emotion" and conducted a systematic review and meta-analysis of the published English literature. We aim to explore the effects of physical activity in and out of the classroom and in and out of school on positive emotions in children and adolescents. We go further to explore the different effects of physical activity interventions on mood by age, the type of exercise, and the duration of exercise.

## 2. Materials and Methods

### 2.1. Search Strategy

We followed the criteria of systematic evaluation and meta-analysis guidelines [25]. In August 2022, we comprehensively searched for original research articles published post-1980 in major databases, namely, Scopus, PubMed, Web of Science, EBSCOhost, and APA PsycInfo; the search was repeated in October 2022 to update the review. The search terms included a combination of the following 3 terms: (1) adolescent * OR teen * OR teenager * OR student * OR juvenile OR “school-aged children”; (2) “physical activity” OR “physical exercise” OR “sports activities” OR “sport movement” OR sport * OR motor OR “athletic sports” OR “aerobic exercise” OR “aerobic training” OR “resistance exercise” OR “strength training” OR “muscle-strengthening exercise” OR “physical education” OR “fitness game”; (3) mood * OR affect* OR emotion * OR happiness OR pleasure OR enjoyment OR subjective well-being OR self-esteem (see Supplementary Materials (Table S1) for the search strategy used for each database). The references included in the literature and in the relevant reviews were also retrospectively reviewed to ensure a complete search. The retrieved literature was sequentially filtered by title and abstract. Then, the full text was extracted for evaluation. Finally, the references of the included studies and relevant studies by experts in the field were manually searched to supplement the missing literature in the electronic literature database. The search was performed independently by two researchers. A third researcher was consulted in case of disagreement.

### 2.2. Study Selection

The inclusion criteria of the relevant studies were based on Participant/Intervention/Comparison/Outcomes/Study Design (PICOS) principles as follows: Participant (P): relevant studies in children and adolescents; Intervention (I): interventions in the form of physical activity, such as aerobic exercise, fitness exercises, and physical education; Comparison (C): there were no exercise intervention or stretching/relaxation exercises for the control group; Outcomes (O): the main outcome indicator of the study was the positive mood of the children and adolescents; Study Design (S): the study design was an interventional study with a control group (including randomized controlled trials and non-randomized controlled trials).

The exclusion criteria for the relevant studies were as follows: (1) non-English, unpublished literature, conference proceedings, theses, dissertations, and literature reviews; (2) studies of infants and children, the elderly, animals, and specific populations (such as people with disabilities and psychiatric patients); (3) unable to extract valid data from the literature; (4) duplicate published literature; and (5) inaccessible full text.

### 2.3. Data Extraction and Quality Assessment

Two researchers separately performed data extraction and quality scoring for the included studies. Inconsistent results were discussed until an agreement was reached. The data included the first author, publication year, participant characteristics (sample size, age, country, and region), sampling type, research design, interventions, testing tool, and outcome indicators (Table S2).

We evaluated the included literature using the PEDro scale, a credit rating scale developed by the Australian Centre for Evidence-Based Practice [26]. The scale has ten criteria. It includes random grouping (two items), blind method (three items), data reporting items (three items), data analysis (one item), and follow-up (one item). We recorded each item as 1 point when it appeared in an article and 0 points when it was not reflected, with a total score of 0–10 points. Avoiding subjective opinions, two reviewers evaluated the opinions, and a third reviewer judged the differences. The scale was scored out of 10, with scores ≥5 indicating high quality and <5 indicating inferior quality.

### 2.4. Statistical Analysis

A meta-analysis was performed using RevMan 5.4 and Stata 16.0 software. Effect sizes were combined by adopting standardized mean differences (SMDs) and 95% confidence intervals (CIs). Before the meta-analysis, a heterogeneity test was performed, and the heterogeneity between the included studies was tested using *I*^2^. *I*^2^ can quantify the magnitude of heterogeneity. The value range is 0–100%, and the larger the *I*^2^ value, the more significant the heterogeneity among the studies. If *I*^2^ ≤ 50%, it means that the statistical heterogeneity among the studies is small, and the fixed effects model was used for the meta-analysis; if *I*^2^ > 50%, it means that there is significant statistical heterogeneity among the studies, and the randomized-effects model was used for the meta-analysis [27]. Moreover, studies with heterogeneity were subjected to subgroup and sensitivity analyses to explore the sources of heterogeneity [28]. Funnel plot, Begg’s and Egger’s tests were used to detect any publication bias [29].

## 3. Results

### 3.1. Literature Screening Process and Results

**Figure 1:**
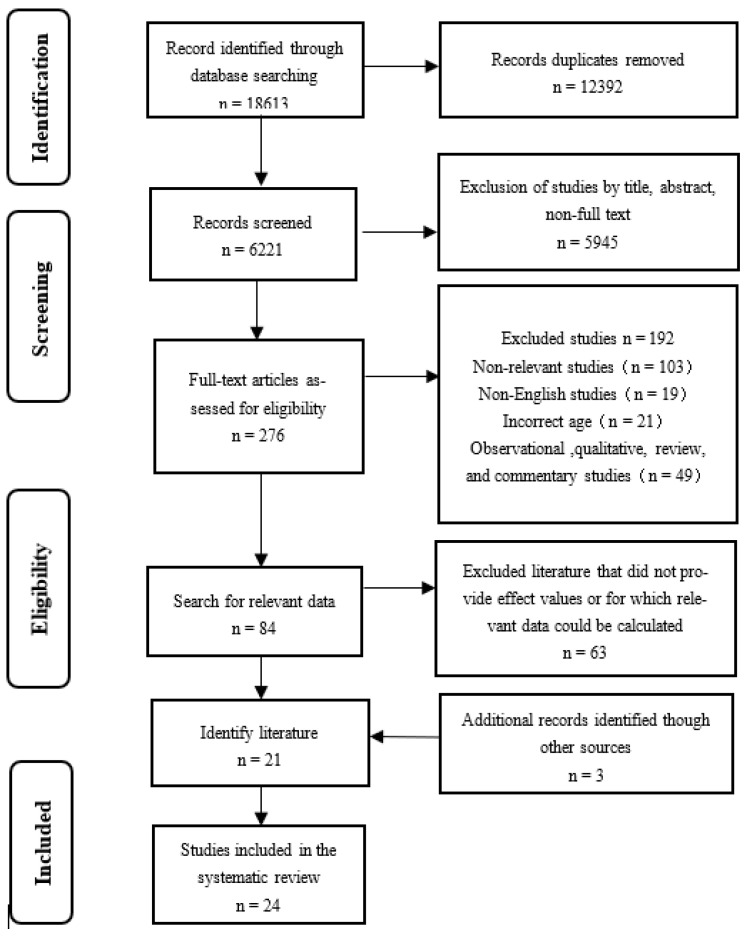
Flow chart of literature retrieval.

After searching for the subject terms, 18613 articles were retrieved from the electronic database. First, the retrieved literature was imported into the literature management software Endnote, and 6221 articles were obtained after removing duplicates. A total of 276 articles were initially obtained after reading the title and abstract and after excluding non-full-text articles. Subsequently, the remaining articles were reviewed by full-text reading, and 84 full-text articles were obtained after excluding the non-relevant literature. Effect values and 95% CIs or relevant data that could be calculated were obtained, and 21 articles were identified. Three additional relevant articles were added through a manual search of the references of the included articles and relevant studies by senior experts in the field. A total of 24 articles were finally included in the meta-analysis of interventional studies. The specific search steps are shown in Figure 1.

### 3.2. Characteristics and Evaluation of the Quality of the Literature

The basic characteristics of the included articles are shown in Table 1. The 24 studies included in the meta-analysis were published between 2007 and 2021, with 3907 respondents from 14 countries. The United States published five studies; Spain and Germany published three studies each; Switzerland and South Korea published two studies each; and Israel, Turkey, Ukraine, Portugal, Britain, Lithuania, Brazil, Canada, and Saudi Arabia published one study each. There were seventeen articles with a sample size of less than 100, accounting for 71% of the total included articles, and there were seven articles with a sample size greater than 100, accounting for 29% of the total included articles. Interventions: A variety of physical activities were used in at least one trial group, and no exercise intervention or stretching and relaxation exercises were used in the control group. Measurements were recorded at each center by referencing or by developing questionnaires and scales based on specific content to measure relevant indicators (Table S2). Subjects ranged in age from 7 to 21 years, with three studies targeting elementary school students (grades 2–6), fifteen targeting middle- and high-school students, and the remaining six targeting first-year college students. Twenty-four studies were included, 12 were of high quality and 12 were of low quality (Table 2).

### 3.3. Meta-Analysis Results

#### 3.3.1. The Relationship between Participation of Children and Adolescents in PA and Positive Emotions

**Figure 2:**
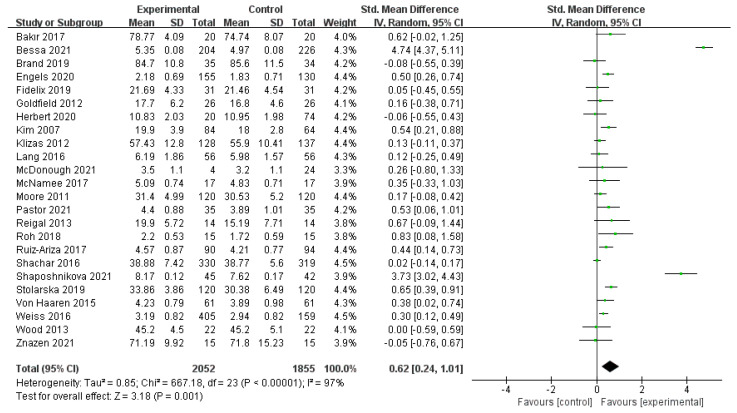
Forest plot of the association between PA and positive emotions in children and adolescents [30,31,32,33,34,35,36,37,38,39,40,41,42,43,44,45,46,47,48,49,50,51,52,53].

Twenty-four studies [30,31,32,33,34,35,36,37,38,39,40,41,42,43,44,45,46,47,48,49,50,51,52,53] reported a relationship between participation in physical activity and emotional change in children and adolescents using positive emotions as an outcome indicator. First, these 24 studies were tested for heterogeneity. There was significant heterogeneity between studies (*I*^2^ = 97% > 50%, *p* < 0.01), and a random-effects model was selected to combine the results. The meta-analysis in Figure 2 shows that positive emotions were significantly better in the physical activity participation group than in the control group without physical activity (SMD = 0.62, 95% CI: (0.24, 1.01), *p* < 0.01). The difference was statistically significant, showing that participation in physical activity significantly causes positive emotions in children and adolescents. We conducted subgroup analyses of the factors that may cause heterogeneity: the age of the participants, the type of exercise, and the duration of the exercise.

#### 3.3.2. Subgroup Analysis

##### Age Factor

**Figure 3:**
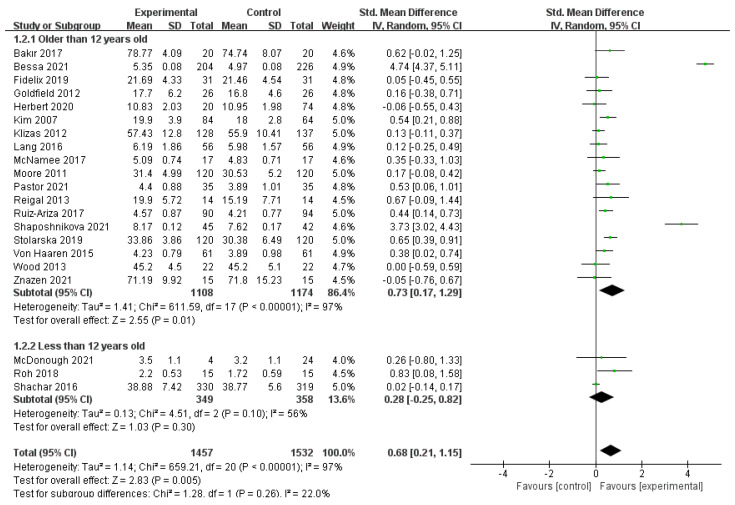
Age subgroup of the association between PA and positive emotions in children and adolescents [30,31,32,33,34,35,36,37,39,40,41,42,43,45,46,47,49,50,51,52,53].

A total of 21 studies, comprising 2989 participants, were included [30,31,32,33,34,35,36,37,39,40,41,42,43,45,46,47,49,50,51,52,53]. The results of the random-effect model meta-analysis are shown in Figure 3. Among the different age subgroups, the total effect value of the 18 studies with a subgroup aged >12 years was SMD = 0.73, 95% CI: (0.17, 1.29), *p* = 0.01, and the pooled effect value of the 3 studies with a subgroup aged ≤12 years was SMD = 0.28, 95% CI: (−0.25, 0.82), *p* = 0.30. The results show that physical activity significantly improved positive emotions in the subgroup aged >12 years compared with the no-exercise group. In the subgroup aged ≤12 years, no significant difference was observed between the physical activity group and the no-exercise group. The between-group difference test of the subgroups showed no difference between the two different age subgroups (*p* = 0.26). The test of heterogeneity suggested that the age of the participants may be a source of heterogeneity.

##### Type of Exercise

**Figure 4:**
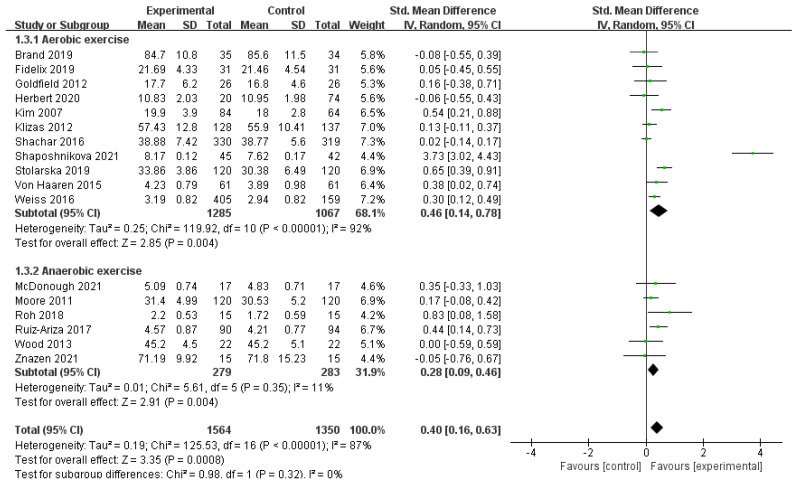
Exercise type subgroup of the association between PA and positive emotions in children and adolescents [30,31,32,33,34,36,38,39,41,43,44,45,46,47,49,50,51].

A total of 17 studies, comprising 2914 participants, were include [30,31,32,33,34,36,38,39,41,42,43,44,45,46,47,49,50]. The results of the random-effects model meta-analysis are shown in Figure 4. In the subgroup of exercise type, the pooled effect value of the 11 aerobic exercise studies was SMD = 0.46, 95% CI: (0.14, 0.78), *p* < 0.01, and the pooled effect value of the six anaerobic exercise studies was SMD = 0.28, 95% CI: (0.09, 0.46), *p* < 0.01. In the aerobic exercise subgroup, the effect of physical activity on positive emotions was higher than in the no-exercise group. In the subgroup of anaerobic exercise, the effect of physical activity on positive emotions was also higher than in the no-exercise group. The between-group difference test of the subgroups showed no significant difference between the subgroups of aerobic and anaerobic exercise (*p* = 0.32). In addition, the heterogeneity test suggested that the type of exercise may be a source of heterogeneity.

##### Duration of Exercise

**Figure 5:**
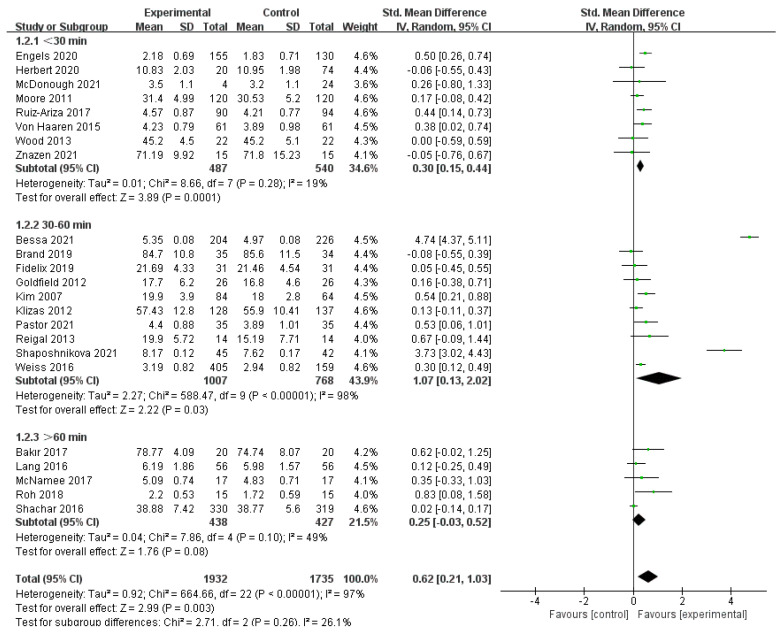
Exercise Duration Subgroup of the Association between PA and Positive Emotions in Children and Adolescents [30,31,32,33,34,35,36,37,38,39,40,41,42,43,44,46,47,48,49,50,51,52,53].

A total of 23 studies, comprising 3667 participants, met the inclusion criteria [30,31,32,33,34,35,36,37,38,39,40,41,42,43,44,46,47,48,49,50,51,52,53]. The results of the random-effects model meta-analysis are shown in Figure 5. Among the subgroups with different exercise durations, 10 studies with an exercise duration of 30-60 min had a pooled effect value of SMD = 1.07, 95% CI: (0.13, 2.02), *p* < 0.05; 8 studies with an exercise duration of <30 min had a pooled effect value of SMD = 0.30, 95% CI: (0.15, 0.44), *p* < 0.01); and 5 studies with an exercise duration of ≥60 min had a pooled effect value of SMD = 0.25, 95% CI: (−0.03, 0.52), *I*^2^ = 49%, *p* = 0.08. In the 30–60 min subgroup, the improvement in positive emotions was significantly greater in the physical activity group than in the other groups. The between-group difference test of the subgroups showed no significant difference between the subgroups (*p* = 0.26). The test of heterogeneity suggested that the duration of exercise may be a source of heterogeneity.

#### 3.3.3. Sensitivity Analysis

**Figure 6:**
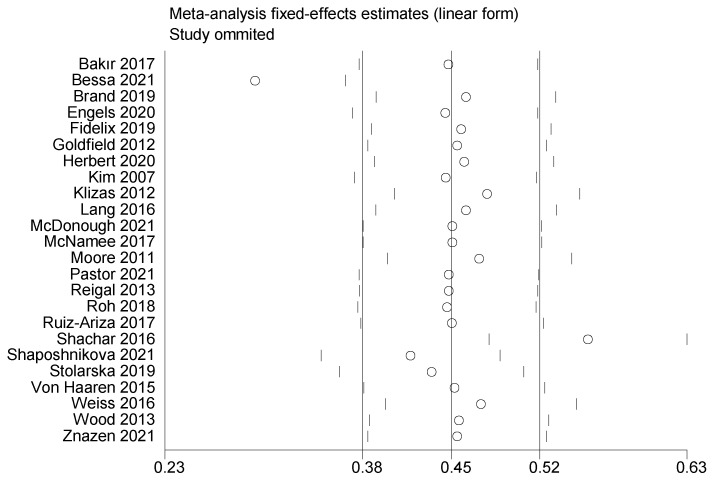
Sensitivity analysis of the association between PA and positive emotions in children and adolescents [30,31,32,33,34,35,36,37,38,39,40,41,42,43,44,45,46,47,48,49,50,51,52,53].

To further explore the source of heterogeneity, a sensitivity analysis was performed on the studies. Individual studies were excluded in turn. The results of the analysis are shown in Figure 6. Consistent with the original analysis results, the individual studies had little effect on the combined results. This indicates that the combined effect values of this study were more stable.

#### 3.3.4. Publication Bias

**Figure 7:**
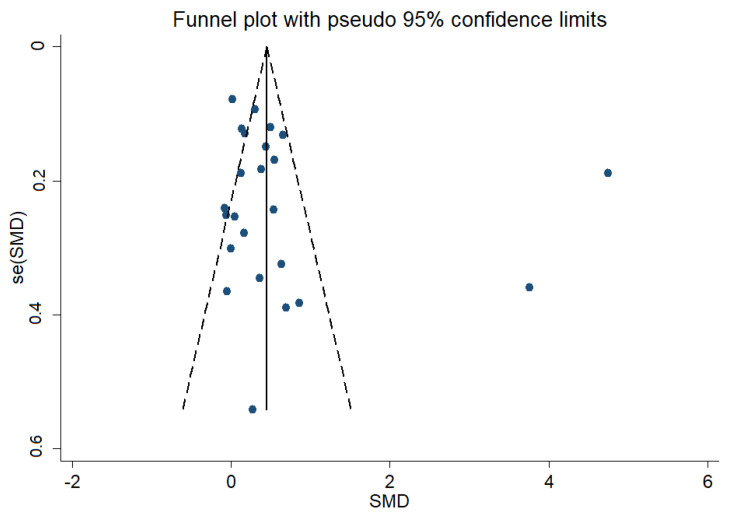
Funnel graph of publication bias of the association between PA and positive emotions in children and adolescents.

In this study, a publication bias test was performed on the included literature. As shown in Figure 7, there is asymmetry at the bottom right of the funnel plot. This predicts the possible existence of publication bias. However, this method is mainly based on subjective judgment and may be inaccurate. Therefore, Begg’s (Z = 1.84, *p* = 0.07) and Egger’s (T = 1.07, *p* = 0.29) tests were further applied, and the results show that *p* > 0.05. This indicates that there was no significant publication bias in the literature.

## 4. Discussion

This study presents a comprehensive quantitative analysis of several independent studies related to physical activity and positive emotions in children and adolescents through a meta-analysis. A meta-analysis helps us to expand the total sample size and reduce the selection bias of the study subjects, thus compensating for the poor statistical efficacy and bias in individual studies and making the conclusions more convincing [54]. The meta-analysis results showed that the participation of children and adolescents in physical activity was positively associated with positive emotions. This suggests that active participation in sports by children and adolescents improves emotional well-being. This finding is similar to that of a review of the relationship between physical activity and emotions conducted by Chan et al. (2019) [55]. The medical field currently holds four different explanations for how physical activity affects the emotions of children and adolescents. One view considers distraction and suggests that children and adolescents distracted from unfavorable stimuli while participating in physical activity experience significant improvements in emotions during and after the activity [56]. Another view considers self-efficacy and suggests that physical activity could be seen as a challenging activity. Regular physical activity may help improve emotions and increase confidence [57]. A third view considers social interaction and refers to the existence of the social relationships inherent in physical activity. The mutual support between individuals involved in physical activity plays an important role in influencing positive emotions [58]. In addition, physiological explanations suggest that participation in physical activity increases the synaptic transmission of monoamines and activates the secretion of endorphins [59]. These substances have an inhibitory effect on the central nervous system. This reduces pain and enhances the active state of the brain, resulting in mood improvement after exercise [60]. However, there is no agreement on the above points. Specifically, knowledge about the relationship between physical activity and positive emotions is still limited. Therefore, it is impossible to determine causality or describe the psychological and physiological mechanisms behind this association. The 24 studies included in this meta-analysis review were all intervention studies with a large sample size (3907 people). Each study had a clear description of the selection of the study population. Both the interventions and control groups were from the same populations, and the study populations were well-represented. An examination of the heterogeneity of the included studies showed that there was significant heterogeneity among the studies. Therefore, this study further explored the potential influencing factors and sources of heterogeneity.

### 4.1. The Effects of Physical Activity on the Emotions of Children and Adolescents of Different Ages

The subgroup analysis revealed that, in the subgroup of subjects older than 12 years, the emotional improvement of the physical activity group was significantly higher than that of the non-exercise group. The effect of physical activity on positive emotions was higher in the subgroup aged over 12 years than in the subgroup aged ≤12 years. The result is consistent with Zimmermann’s findings. The review by Zimmermann (2020) suggested that children and adolescents over 12 years gradually develop more effective emotional regulation [61]. The reason for this may be the gradual improvement of the nervous system and brain structure as children grow older [62,63]. Children and adolescents are more capable of paying attention to and reflecting on their internal emotional states [64]. This is an interesting finding. However, this result needs to be interpreted with caution due to the small number of included studies with a subgroup aged ≤12 years, which may have affected the effect size statistics. Future studies need to explore the intervention effects of physical activity participation on positive emotions in children and adolescents of different ages.

### 4.2. The Effects of Exercise Type on the Emotions of Children and Adolescents

The subgroup analysis showed that positive emotions were found to be significantly higher in the physical activity group than in the no physical activity group in both aerobic and anaerobic subgroups. The improvement in positive emotions was better in the aerobic subgroup. The research showed that participation in physical activity triggers emotional responses that are determined by a combination of cognitive factors (e.g., physical self-efficacy) and signals from visceral receptors [65]. When exercise intensity exceeds the ventilatory threshold (VT), visceral organ perception significantly affects the emotional experience of the physically active person. Thus, aerobic exercise below the ventilatory threshold could trigger positive emotional experiences, and anaerobic exercise above the ventilatory threshold may reduce positive emotional experiences. This is consistent with Rethorst’s (2009) meta-analysis, which concluded that aerobic exercise has a therapeutic effect on emotions [66]. However, a study by Ramalho et al. found no significant difference between the effect of aerobic and anaerobic exercise on emotions [67]. Currently, whether there are differences between the effects of different types of physical activities on the positive emotions of children and adolescents cannot be concluded. The effects of different types of physical activities on interventions for the positive emotions of children and adolescents need to be further explored in future studies.

### 4.3. The Effects of Exercise Duration on the Emotions of Children and Adolescents

The subgroup analysis showed that the effect of participation in physical activity on positive emotions was significantly higher in the subgroup with a 30–60 min exercise duration than in the other two groups. The small number of included studies may have affected the effect size statistics. The current evidence suggests that exercise duration may be a source of heterogeneity. Further studies with large samples are needed to verify the validity of this result. Some studies have pointed out that exercise for too short a time may not achieve the effect of emotional relief [68]. It has also been suggested that prolonged exercise may trigger androgenic–anabolic steroids (AAS) [69]. The effects of these substances manifest as a significant increase in irritability and aggression, potentially triggering negative emotions. Therefore, the exercise duration for children and adolescents should be limited to more than 30 min but less than 60 min as much as possible. This finding coincides with that of the study of Peluso (2005) [70], and it is promising.

## 5. Conclusions and Future Prospects

This meta-analysis demonstrated that the participation of children and adolescents in physical activity was positively associated with positive emotions. The age, type of exercise, and duration of the exercise of children and adolescents may be important factors influencing the positive emotions resulting from physical activity interventions. The physical activity group was significantly better than the control group without physical activity in terms of positive emotions. The participation of adolescents aged ≥12 years in aerobic exercise for 30–60 min had a more significant effect on positive emotions. Based on the results, we hope that schools will optimize their curricula and add more time for physical activity, keeping aerobic activity to 30–60 min for youth over the age of 12. Parents should assist schools in encouraging children and adolescents to participate in physical activities together in order to promote physical health and improve mental health at the same time. Our goal is to help children and youth grow up happily and healthily by improving their positive emotions through physical activity interventions. 

The findings of this study further suggest that follow-up research on the effects of physical activity on positive emotions in children and adolescents requires in-depth refinement. More high-quality studies, especially randomized controlled studies, are needed to verify the reliability of the results in a follow-up. In terms of research content, there is a need to explore the variability of the effects of physical activity on emotions by gender, age group, type of exercise, the intensity of exercise, and place of activity. In addition, for the measurement of relevant indicators, the current study mainly used subjective questionnaires or scales, which may be biased. Therefore, follow-up studies may consider the use of advanced emotion sensors for the quantitative detection of the emotions of children and adolescents.

## Figures and Tables

**Table 1:** Study Characteristics.

Author (Year)	Country	Sample Characteristics	Sampling Type	Research Design	Intervention Measure	Intervention Time	Testing Tool
Kim 2007 [30]	South Korea	High-school students (n = 45) and college students (n = 232), average age of 20.6 years	Intervention research	Randomly assigned	Aerobic exercise, physical conditioning, street dance, and skating	40 min	The Subjective Exercise Experiences Scale
Moore 2011 [31]	United States of America	120 college students (83 males and 37 females), average age of 20.2 years	Intervention research	Non-random allocation	Resistance movement	12 weeks	Rosenberg Self-Esteem Scale (RSE)
Klizas 2012 [32]	Lithuania	265 girls aged 14–15	Intervention research	Randomly assigned	Basketball, football, and volleyball	30–35 min, twice a week for 7 months	The life satisfaction scale
Goldfield 2012 [33]	Canada	30 obese teenagers aged 12–17	Intervention research	Randomly assigned	Aerobic exercise	40 min, twice a week for 10 weeks	Self-Perceptions Profile for Adolescents (SPPA)
Wood 2013 [34]	Britain	25 teenagers, average age of 13.1 years	Intervention research	Randomly assigned	Constant load test	15 min	The Adolescent Profile of Mood States (POMS-A) Questionnaire
Reigal 2013 [35]	Spain	67 teenagers aged 14–17	Intervention research	Non-random allocation	Team physical education class	45 min	Profile of Mood States (POMS) Questionnaire
Von Haaren 2015 [36]	Germany	61 students, average age of 21.6 years	Intervention research	Randomly assigned	Aerobic exercise	20 min, twice a week for 20 weeks	Multidimensional Mood Questionnaire (MDMQ)
Lang 2016 [37]	Switzerland	31 students, average age of 16.2 years	Intervention research	Randomly assigned	Coping training program in routine physical education class	90-minute physical education class at least once a week	The Adolescent Stress Questionnaire (ASQ)
Weiss 2016 [38]	United States of America	405 students (male 301, female 104), average age of 12.6 years	Intervention research	Non-random allocation	First Tee course	40 min for a year	Self-Perception Profile for Adolescents (SPPA)
Shachar 2016 [39]	Israel	69 children (grades 3–6)	Intervention research	Randomly assigned	Football, basketball, and volleyball	A total of 120 hours of sports activities within 24 weeks	Positive and Negative Affect Schedule
Bakır 2017 [40]	Turkey	60 students in grade 10 of senior high school	Intervention research	Non-random allocation	Organized sports and social activities	2 h a week for 10 weeks	The Positivity Scale
Ruiz-Ariza 2017 [41]	Spain	84 teenagers aged 12–16	Intervention research	Randomly assigned	High-intensity interval training (C-HIIT)	16 min	The Trait and Emotional Intelligence Questionnaire Short Form (TEIQue-SF)
McNamee 2017 [42]	United States of America	7 students, average age of 14.6 years	Intervention research	Non-random allocation	cardiovascular activities muscular strength and endurance activities flexibility activities.	90 min for 4 weeks, 35 classes	Self-Efficacy Questionnaire (SEQ)
Roh 2018 [43]	South Korea	30 children (grades 4–6)	Intervention research	Randomly assigned	TKD training	60 min each time for 16 weeks	The Korean Version of the Profile of Mood State-Brief (K-POMS-B)
Brand 2019 [44]	Switzerland	106 teenagers aged 10–16	Intervention research	Randomly assigned	Aerobic exercise	35 min	A computerized test
Stolarska 2019 [45]	United States of America	120 female college students, average age of 20.60 years	Intervention research	Non-random allocation	aerobic exercise	45 min of aerobic exercise	UWIST Mood Adjective Check List
Fidelix 2019 [46]	Brazil	62 teenagers, average age of 15.0 years	Intervention research	Randomly assigned	Aerobic training	30 min of aerobic training, three times a week for 24 weeks	Rosenberg Self-Esteem Scale
Herbert 2020 [47]	Germany	185 students, 18 years old	Intervention research	Randomly assigned	Aerobic exercise	16 min for six weeks	The Positive and Negative Affect Schedule
Engels 2020 [48]	Germany	285 Junior high school students, average age 12.67, 48.4% female	Intervention research	Randomly assigned	Cooperative game	15-minute cooperative game, once a week, 7–14 weeks	Questionnaire for the Assessment of Enjoyment in Physical Education (QUAEPE)
Shaposhnikova 2021 [49]	Ukraine	87 boys majoring in football	Intervention research	Non-random allocation	Football training	45 min for a year	WAM methodology
Znazen 2021 [50]	Saudi Arabia	46 female students majoring in physical education (average age of 20.02 years)	Intervention research	Randomly assigned	Moderate strength training, with training intensity of 55%, and high-intensity strength training, with training intensity of 80%	Three groups of 8–10 repetitions and three groups of 6 repetitions, each lasting about 30 min	BRUMS
McDonough 2021 [51]	United States of America	47 minority teenagers, average age of 11.8 years	Intervention research	Non-random allocation	Fitness game	Single 15 min	Self-efficacy instrument
Bessa 2021 [52]	Portugal	430 senior high school students, average age of 16.22 years	Intervention research	Non-random allocation	Sport education	one class lasting 45 min and the other class lasting 90 min for 8 weeks.	Competitive State Anxiety Inventory-2 (CSAI-2)
Pastor 2021 [53]	Spain	35 senior high school students, average age of 16.49 years	Intervention research	Randomly assigned	Light/moderate physical activity and moderate and vigorous-intensity physical activity	30 min, once a week, 3 weeks	Short form of the Positive and Negative Affect Schedule

**Table 2:** Quality Assessment of included studies.

Reference	Eligibility Criteria	Random Allocation	Concealed Allocation	Groups Similar at Baseline	Participants Blinded	Provider Blinded	Evaluator Blinded	Follow-Up	Intention-to-Treat Analysis	Between-Group Comparison	PEDro Score
Kim 2007 [30]	0	1	0	0	1	0	0	0	1	1	4
Moore 2011 [31]	1	0	0	0	0	0	0	0	1	1	3
Klizas 2012 [32]	0	1	0	1	0	0	0	0	1	1	4
Goldfield 2012 [33]	1	1	1	1	0	0	0	1	1	1	7
Wood 2013 [34]	0	1	1	1	1	0	0	0	1	1	6
Reigal 2013 [35]	0	0	0	0	0	0	0	0	1	1	2
Von Haaren 2015 [36]	1	1	0	1	0	0	0	0	1	1	5
Lang 2016 [37]	1	1	1	1	1	1	0	1	1	1	9
Weiss 2016 [38]	0	0	0	1	0	0	0	1	0	1	3
Shachar 2016 [39]	1	1	0	1	0	0	0	0	1	1	5
Bakır 2017 [40]	0	0	0	1	1	0	0	0	0	1	3
Ruiz-Ariza 2017 [41]	1	1	1	1	1	1	0	0	1	1	8
McNamee 2017 [42]	0	1	0	0	0	0	0	0	1	1	3
Roh 2018 [43]	1	1	0	1	0	0	0	0	1	1	5
Brand 2019 [44]	1	1	0	0	1	0	0	0	1	1	5
Stolarska 2019 [45]	1	0	0	1	0	0	0	0	1	1	4
Fidelix 2019 [46]	1	1	1	1	0	0	0	1	1	1	7
Herbert 2020 [47]	1	1	1	1	1	0	0	0	1	1	7
Engels 2020 [48]	0	1	0	0	0	0	0	0	1	1	3
Shaposhnikova 2021 [49]	0	0	0	1	0	0	0	0	0	1	2
Znazen 2021 [50]	1	1	1	1	0	0	0	0	1	1	6
McDonough 2021 [51]	0	0	0	0	1	0	0	0	1	1	3
Bessa 2021 [52]	0	0	0	0	0	0	0	0	1	1	2
Pastor 2021 [53]	1	1	0	0	0	0	0	1	1	1	5

## Data Availability

Not applicable.

## Supplementary Materials

The following supporting information can be downloaded at: https://www.mdpi.com/article/10.3390/ijerph192114185/s1, Table S1: Search strategies. Table S2: The testing tools.
