# Supplementary material for: The Effects of Physical Activity on Positive Emotions in Children and Adolescents: A Systematic Review and Meta-Analysis
Source: Int J Environ Res Public Health. 2022 Oct 30;19(21):14185. doi: 10.3390/ijerph192114185 (PMC9657539; doi:10.3390/ijerph192114185)
Supplement: Supplementary file 1 [file ijerph-19-14185-s001.zip › ijerph-1941023-supplementary.pdf]

Table S1: Search strategies.

Web of science=2782	((TI=(adolescent* OR teen* OR teenager* OR student* OR juvenile OR "school-aged children")) AND TI=("physical activity" OR "physical exercise" OR "sports activities" OR "sport movement" OR sport* OR motor OR "athletic sports" OR "aerobic exercise" OR "aerobic training" OR "resistance exercise" OR "strength training" OR "muscle-strengthening exercise" OR "physical education" OR "fitness game")) AND TS=(mood* OR affect* OR emotion* OR happiness OR pleasure OR enjoyment OR subjective well-being OR self-esteem)
Scopus=1974	(TITLE-ABS-KEY (adolescent* OR teen* OR teenager* OR student* OR juvenile OR "school-aged children") AND TITLE-ABS-KEY ("physical activity" OR "physical exercise" OR "sports activities" OR "sport movement" OR sport* OR motor OR "athletic sports" OR "aerobic exercise" OR "aerobic training" OR "resistance exercise" OR "strength training" OR "muscle-strengthening exercise" OR "physical education" OR "fitness game") AND TITLE-ABS-KEY (mood* OR affect* OR emotion* OR happiness OR pleasure OR enjoyment OR subjective well-being OR self-esteem))
Pub-med=6808	((adolescent*[Title/Abstract] OR teen*[Title/Abstract] OR teenager*[Title/Abstract] OR student*[Title/Abstract] OR juvenile[Title/Abstract] OR "school-aged children"[Title/Abstract]) AND ("physical activity"[Title/Abstract] OR "physical exercise"[Title/Abstract] OR "sports activities"[Title/Abstract] OR "sport movement"[Title/Abstract] OR sport*[Title/Abstract] OR motor[Title/Abstract] OR "athletic sports"[Title/Abstract] OR "aerobic exercise"[Title/Abstract] OR "aerobic training"[Title/Abstract] OR "resistance exercise"[Title/Abstract] OR "strength training"[Title/Abstract] OR "muscle-strengthening exercise"[Title/Abstract] OR "physical education"[Title/Abstract] OR "fitness game"[Title/Abstract])) AND (mood*[Title/Abstract] OR affect*[Title/Abstract] OR emotion*[Title/Abstract] OR happiness[Title/Abstract] OR pleasure[Title/Abstract] OR enjoyment[Title/Abstract] OR subjective well-being[Title/Abstract] OR self-esteem[Title/Abstract])
EBSCOhost ERIC=2046	AB (adolescent* OR teen* OR teenager* OR student* OR juvenile OR "school-aged children") AND AB ("physical activity" OR "physical exercise" OR "sports activities" OR "sport movement" OR sport* OR motor OR "athletic sports" OR "aerobic exercise" OR "aerobic training" OR "resistance exercise" OR "strength training" OR "muscle-strengthening exercise" OR "physical education" OR "fitness game") AND AB (mood* OR affect* OR emotion* OR happiness OR pleasure OR enjoyment OR subjective well-being OR self-esteem)
APA PsycInfo =5003	AB (adolescent* OR teen* OR teenager* OR student* OR juvenile OR "school-aged children") AND AB ("physical activity" OR "physical exercise" OR "sports activities" OR "sport movement" OR sport* OR motor OR "athletic sports" OR "aerobic exercise" OR "aerobic training" OR "resistance exercise" OR "strength training" OR "muscle-strengthening exercise" OR "physical education" OR "fitness game") AND AB (mood* OR affect* OR emotion* OR happiness OR pleasure OR enjoyment OR subjective well-being OR self-esteem)

Table S2 The testing tools.

Author / Year	Tool	Tool Description
Kim 2007	The Subjective Exercise Experiences Scale	The scale has 12 items which represent three dimensions of positive well-being (e.g., positive affect), psychological distress (e.g., anxiety, depression, stress related emotions), and fatigue.
Moore 2011	Rosenberg Self-Esteem Scale (RSE)	The RSE is a 10-item scale consisting of general statements about oneself that are scored 1–4 and summed to produce a self-esteem score with a potential range of 10–40.
Klizas 2012	The life satisfaction scale	The multidimensional students' life satisfaction scale is a 7-item self-report inventory. The subjects had to choose one answer to evaluate each statement as "strongly disagree," "disagree," "neither disagree nor agree," "agree," and "strongly agree.".
Goldfield 2012	Self-Perceptions Profile for Adolescents (SPPA)	This inventory is designed to measure adolescents' perceived competence in six domains of functioning: Scholastic ability (5 items), Social competence (10 items), Athletic competence (5 items), Physical appearance (5 items), Behavioral conduct (6 items), and Global self-esteem (4 items).
Wood 2013	The Adolescent Profile of Mood States Questionnaire (POMS-A)	The questionnaire is mainly used to monitor the Adolescents' mood.
Reigal 2013	Profile of Mood States questionnaire (POMS)	This instrument assesses six states, namely, tension-anxiety, depression-dejection, anxiety-hostility-anger, vigor-activity, fatigue-inertia and confusion-bewilderment.
Von Haaren 2015	Multidimensional Mood Questionnaire (MDMQ)	MDMQ is validated especially for Ambulatory Assessment. The scale measures three basic mood dimensions, valence, calmness and energetic arousal.
Lang 2016	The Adolescent Stress Questionnaire (ASQ)	The ASQ measures the perceived stressfulness of events that adolescents commonly experience in their daily lives.
Weiss 2016	Self-Perception Profile for Adolescents (SPPA)	These subscales tap youths' beliefs about doing well in classwork and feeling accepted by peers. Each subscale consists of five items, and responses are given in a structured-alternative format.
Shachar 2016	Positive and Negative Affect Schedule	This is a self-report checklist of affect adjectives, was designed to provide independent measures of positive and negative affect.
BakırYakup 2017	The Positivity Scale	The positivity scale consists of eight items, one of which is reverse-scored. Minimum 8 and maximum 40 points can be taken from the measurement tool.

Ruiz-Ariza 2017	The Trait and Emotional Intelligence Questionnaire Short Form (TEIQue-SF)	TEIQue-SF is composed of 30 items with seven possible responses to each statement ranging from 'Completely disagree' ¼ 1 to 'Completely agree' ¼ 7.
McNamee 2017	Self-Efficacy Questionnaire (SEQ)	The Self-Efficacy Questionnaire was used to assess confidence in participants' ability to be physically active. The eight items on the SEQ were rated on a 5-point scale ranging from 1 (disagree a lot) to 5 (agree a lot), with higher scores reflecting greater levels of confidence.
Roh 2018	The Korean Version of the Profile of Mood State-Brief (K-POMS-B)	To estimate the mood state, this questionnaire consists of a total of 30 items.
Brand 2019	A computerized test	The test included two levels of task difficulty and two concepts of emotion processing, namely, emotion labeling and emotion matching.
Stolarska 2019	The UWIST Mood Adjective Check List	The list assesses three dimensions, has been utilized in several exercise studies.
Fidelix 2019	Rosenberg Self-Esteem Scale	It was used to measure self-esteem. It has 10 items that assess attitudes and positive or negative sense of self, encompassing issues relating to personal satisfaction, self-deprecation, perception qualities, competence, pride in themselves, self-worth, respect, and sense of failure.
Herbert 2020	The Positive and Negative Affect Schedule	The state scale assesses positive and negative affect at a given point in time.
Engels 2020	Questionnaire for the Assessment of Enjoyment in Physical Education (QUAEPE)	The Questionnaire is to measure the Enjoyment of physical three facets (pleasure, flow experience, recovery).
Shaposhnikova 2021	WAM methodology	The test includes: Well-being, Activity, Mood.
Znazen 2021	BRUMS	This questionnaire contains 24 items divided into six respective subscales: anger, confusion, depression, fatigue, tension, and vigour.
McDonough 2021	Self-efficacy instrument	To assess participants' perceived PA-related self-efficacy immediately after each exergaming session.
Bessa 2021	Competitive State Anxiety Inventory-2 (CSAI-2)	CSAI-2 has been widely used as a means of explaining the independent forms of influence of somatic anxiety, cognitive anxiety, and self-confidence on sport performance.
Pastor 2021	Short form of the Positive and Negative Affect Schedule	The Schedule was used to measure positive and negative feelings before and after the sessions of physical education.
